# Kinetics of BCR-ABL Transcripts in Imatinib Mesylate treated Chronic Phase CML (CPCML), A Predictor of Response and Progression Free Survival

**Published:** 2009-09

**Authors:** Hossam K Mahmoud, Yasser El Nahas, Mohamad Abdel Moaty, Raafat Abdel Fattah, Mohamad El Emary, Wafaa El Metnawy

**Affiliations:** 1 *Department of Hematology and BMT, Cairo University, Egypt*; 2 *Department of Clinical Pathology, National Cancer Institute (NCI), Cairo University, Egypt*; 3 *Department of Hematology, Nasser Institute, Ministry of Health, Egypt*; 4 *Molecular and Cell Biology Unit, Clinical Oncology center, School of Medicine, Cairo university, Egypt*

**Keywords:** CML, imatinib mesylate, molecular responses, cytogenetic responses, ABL kinase mutations

## Abstract

Purpose: To assess the kinetics of molecular response to Imatinib Mesylate (IM) therapy in predicting progression free survival (PFS), sustained hematological, and cytogenetic responses in CPCML. Methods: Ninety five newly diagnosed CPCML Egyptian patients were treated with IM 400 mg daily dose. Cytogenetic analysis was performed at diagnosis and every 6 months. Molecular monitoring by RT-QPCR was performed at diagnosis and every 3 months during a median follow-up period (FUp) of 26 months. Mutation detection of ABL domain was performed by ASO-PCR. Results: Hematological response was 98% after three months of IM therapy. Out of 95 patients 59 showed 2 log reduction of BCR-ABL/ABL ratio after 6 months of whom 49 (83%) had complete cytogenetic response (CCyR) and 42 (71%) had major molecular response (MMR) at 12 months. BCR-ABL transcripts remained undetectable in 22 patients (39%) at 26 months. Among the remaining 34 patients not achieving 2 log reduction at 6 months only 5 (15%) had CCyR and MMR by 12 months. ABL domain mutations were detected in 11/15 (73%) resistant and suboptimal responding patients. Achieving 2 log reduction after 6 months of IM therapy significantly correlated with sustained cytogenetic and molecular responses (*p*<0.0001), with PFS at 2 years (*p*<0.03) and inversely with ABL gene mutations (*p*<0.001). Discussion: These data demonstrated the predictive value of early molecular response to IM in CPCML regarding disease course and PFS. A 2 log reduction at 6 months of IM treatment could be a cut off level predicting resistance, CCyR, or suggesting IM dose modification.

## INTRODUCTION

Chronic Myeloid Leukemia (CML) is a hematopoietic stem cell disorder representing a clinical model for molecular therapy. The reciprocal translocation t(9;22) (q34; q11) resulting in BCR-ABL oncogenic fusion gene or the Philadelphia chromosome (ph’) is expressed as a fusion protein with deregulated tyrosine kinase activity that has been recognized to play a key role in the pathogenesis of the disease (1). The only proven curative therapy for CML is allogeneic stem cell transplantation (allo-SCT), however; this approach is available for only less than 40% of patients who have an HLA matched donor (2).

The introduction of imatinib mesylate (IM) in 1998 has revolutionized the management of CML. The drug is a selective tyrosine kinase inhibitor (3) which acts by occupying the ATP-binding site of the ABL tyrosine kinase component and maintains it in an inactive conformation. There is data which has demonstrated a correlation between the degree of cytogenetic and molecular response, with improved outcome (4–7). The availability of detecting, with high sensitivity, the levels of BCR-ABL transcripts in patients' blood during the course of therapy has enabled a better ability to follow up the clinical and hematological course of the disease during therapy and has significantly impacted on predicting disease free survival (DFS) and overall survival (OS) in patients under treatment (8). It has been shown that BCR-ABL mRNA level is an independent prognostic marker of PFS when measured either at the time of first CCyR or when the molecular response exceeds 3-log reductions from baseline (6). Moreover, a rising level of BCR-ABL transcripts during therapy can predict a risk of losing cytogenetic remission (5, 6).

The aim of this study was:

1. To determine the predictive potential of the kinetics of early molecular responses to IM 400 mg therapy in maintaining a sustained CCyR, MMR and prolonged PFS;
2. To assess the value of regular molecular monitoring in the early management of resistance or suboptimal responses.

## PATIENTS AND METHODS

Ninety five CPCML patients were enrolled in this study. They had regular assessment in the Medical Oncology-Hematology Departments of Nasser Institute Ministry of Health and at the National Cancer Institute, Cairo University. They were all treated with 400 mg IM daily, according to an approved protocol by the Institutions Review board. All patients provided informed consent according to declaration of Helsinki.

Patients' evaluation at diagnosis: included detailed history and physical examination, complete blood counts (CBC) and blood chemistry for liver and kidney functions. All patients had a bone marrow (BM) evaluation for morphology, conventional cytogenetic analysis for ph’ chromosome as well as a pretreatment RT-QPCR for BCR-ABL fusion gene (9). After the start of treatment patients were monitored every 2 weeks for their blood counts during the first three months and performed a BM analysis after 6 and 12 months of therapy for conventional metaphase analysis and/or FISH analysis whenever necessary. During the second year, patients were examined at least twice for BM metaphases by conventional G banding, performing karyotyping according to standard technique (10). FISH analyses were made by separate hybridizations using fluorescent labled probes for BCR and ABL genes (Vysis, UK). Molecular detection of BCR-ABL transcript level was performed on total RNA extract from peripheral blood. RNA was reverse transcribed and RT-QPCR multiplex assay for both ABL and BCR-ABL was performed to detect both the b2a2 and b3a2 transcripts with total ABL as the normalizing transcript (11, 12). The absolute copy numbers of BCR-ABL target sequence and of the ABL control gene were calculated at the end of reactions using a calibration curve prepared from a set of BCR-ABL and ABL RNA plasmid based standards obtained from Ipsogene, France. Results of relative quantitations were then expressed as BCR-ABL/ABL ratio (%). Assays were performed using Taqman probes, (Applied Biosystems) by real-time PCR (Smart Cycler machine, Cepheid, USA). The sensitivity of each run was confirmed to be in range of 1:100000. PCR results were rejected when normalizing ABL transcript in any given sample was <10 000 copies.

Detection of point mutations in the ABL Kinase domain was performed by Allele-Specific Oligonucleotide Polymerase Chain Reaction (ASO-PCR) (12). Extracted Genomic DNA was submitted to ASO-PCR reactions using specific primers. Both mutated and wild type sequences were amplified using allele specific and reverse primers to detect 10 most common mutations Q252H (a & b), Y253H, Y253F, E255K, E255V, P311L, T315I, M351T, E355G and F359V as previously described (13, 14). Amplified products were visualized using ethidium-bromide agarose gel electrophoresis.

### Definition of responses

**Complete hematological Response (CHR):** WBC count <10 × 10^9^/L, platelet count <450 × 10^9^/L, no immature cells (blasts, promyelo and myelocytes) in peripheral blood and disappearance of palpable splenomegaly (9).

**Cytogenetic responses (CyR):** Major (MCyR: 0–35% Ph +ve) Complete (CCyR: 0% Ph +ve), Partial (PCyR: 1–35% Ph +ve) and Minor (36–90% Ph +ve) (15).

**Molecular responses (MR):** Major Molecular response (MMR) was defined as a ≥3 log reductions from standardized baseline value established by the laboratory, maximum measurable molecular response >4.5 log reduction from baseline level and complete molecular response or negativity with complete disappearance of the Ph+ve clone (15).

**Optimal Response:** Defined as CCyR and MMR within 12 Month of IM.

**Suboptimal Response:** defined as less than CHR at 3 m, less than partial cytogenetic response (PCyR) at 6 m, less than CCyR at 12 m, less than MMR at 18 m (9).

**Failure/primary resistance:** was defined as no CHR at 3 month, less than CHR at 6 month, less than PCyR at 12 month, less than CCyR at 18 m and at any time loss of CHR, loss of CCyR or mutation detection (15).

**Disease Progression:** Occurrence of any of the following events after achieving CCyR: death, accelerated phase (AP) or acute blastic crisis (ABC), loss of CHR or CCyR (6).

### Statistical Methods

Statistical analysis system version 10 was used for data analysis. Mean and standard deviation were estimates of quantitative data. Chi-square/Fischer exact tests were tests of proportion independence. Non parametric t-test and non-parametric ANOVA compared 2 and more than 2 independent groups. P value was always 2 tailed and significant at ≤0.05 levels (16).

## RESULTS

Between March 2003 and March 2008, 95 patients with CPCML, 64 males (67%) and 31 females (33%) presented to Hematology Departments of the National Cancer Institute, NCI, Cairo University and Nasser Institute, Ministry of health, Egypt. Median age at diagnosis was 40 years [15–60]. Median TLC was 156.5 × 10^9^/L [19.2–500], median Hb was 11.3 g/dl [8.4–15.5] and median platelet count was 313 × 10^3^/ul [95–1000]. Starting IM dose was 400 mg, increased to 600 or 800 mg in suboptimal and non responders. Following complete diagnostic work up of CML and initiation of IM therapy all patients have been monitored for molecular response in relation to hematological, cytogenetic responses as well as to other parameters of disease status, for a median duration of 26 months (6–64 months). Hematological response was achieved within 3 months of IM monotherapy in 93 of 95 patients (98%).

### Kinetics of Molecular and cytogenetic Responses

Ninety three patients were evaluable for cytogenetic and molecular responses according to definition criteria. The median pretreatment BCR-ABL/ABL ratio before IM was 2.5 [0.2–20]. Table 1 demonstrates the correlation between a 2 log reduction of BCR-ABL transcripts at 6 month of IM treatment and molecular and cytogenetic responses. Fifty nine patients showed a 2 log reduction of BCR-ABL/ABL ratio after 6 months of IM treatment, of whom only 24 patients (26%) had shown CCyR. At 12 months 49 patients (83%) achieved CCyR and 42 patients (71%) achieved MMR. Among the remaining 34 patients who failed to achieve 2 log reduction at 6 months only 5 patients (15%) had CCyR and MMR by 12 months. The differences in cytogenetic and molecular responses in relation to 2 log reduction of BCR-ABL transcripts at 6 months were statistically significant (*p*<0.0001). Patients who failed to achieve 2 log reductions at 6 months (34/93 patients, 36%) were either suboptimal responders (18/93 patients, 19%) or showed primary resistance (16/93 patients, 17%). By increasing IM dose to 600 mg to these patients, 4 more patients attained a CCyR and MMR at 18 months, Table 1. Another five suboptimal responders demonstrated a rise of their BCR-ABL transcripts whereas other five patients were shifted to second generation tyrosine kinase Inhibitors (TKI). Accordingly, MMR was achieved by 6 patients (7%) at 6 months, by 47 patients (50%) at 12 month and by 61 (65%) patients at 18 months. Table 2 demonstrates a significant correlation between the 2 log reduction value at 6 months and response category, as 97% of responders had 2 log reduction of BCR-ABL transcripts versus only 11% and 0% for the suboptimal and non responders, (*p*<0.0001). Median time to achieve MMR was 12 months (range 6–18 month) in optimal responding patients to IM. Median time to achieve CCyR varied from 9 months [6–24] in patients who entered MMR by 6 and 12 months, to 12 months [9–36] in patients who achieved MMR by 18 months (*p*<0.001). Molecular negativity was recorded in 22 patients (24%) of the whole studied group (93 patients) upon two consecutive testings and till time of preparing the manuscript. Primary resistance was observed in 16/93 (17%) patients who did not demonstrate a decrease in their BCR-ABL transcripts or cytogenetic response since the start of treatment.

**Table 1:** Correlation between early reduction of BCR-ABL transcripts and cytogenetic and molecular responses

2 log reduction at 6 Months	CCyR at 6 Months	CCyR at 12 Months	MMR at 12 Months	MMR & CCyR at 18 Months

Total 93 patients				
Yes (no: 59)	24 (26%)	49 (83%)	42 (71%)	52 (88%)
No (no: 34)	0 (0%)	5 (15%)	5 (15%)	9 (26%)
*P*		0.0001	0.0001	0.0001

**Table 2:** Association between patients' categories and achievement of 2 log reductions at 6 months

	Two log reductios at 6 months	
	No	Yes

Optimal responders (n:59)	2 (3%)	57 (97%)
Suboptimal responders (n:18)	16 (89%)	2 (11%)
Primary resistance (n:16)	16 (100%)	0 (0%)

*p*<0.0001

**Figure 1:**
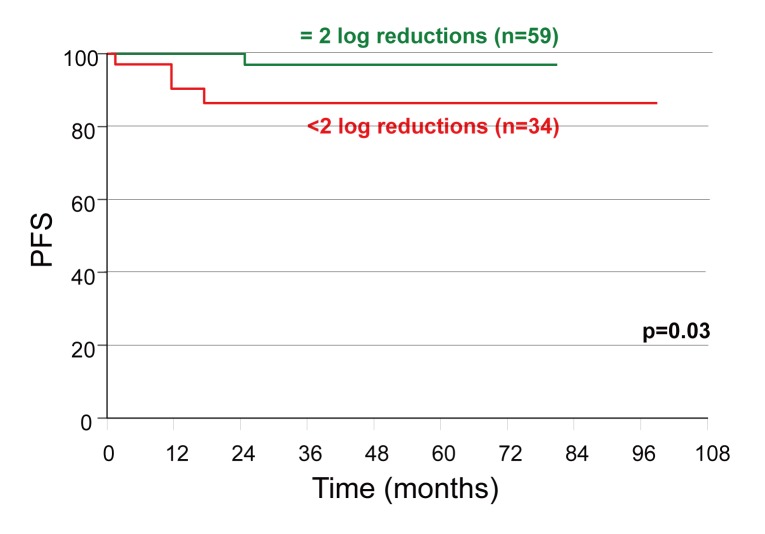
Two log reductions in Bcr-Abl/Abl mRNA ratio at 6 months predicts better PFS at 24 months of IM therapy.

**Figure 2:**
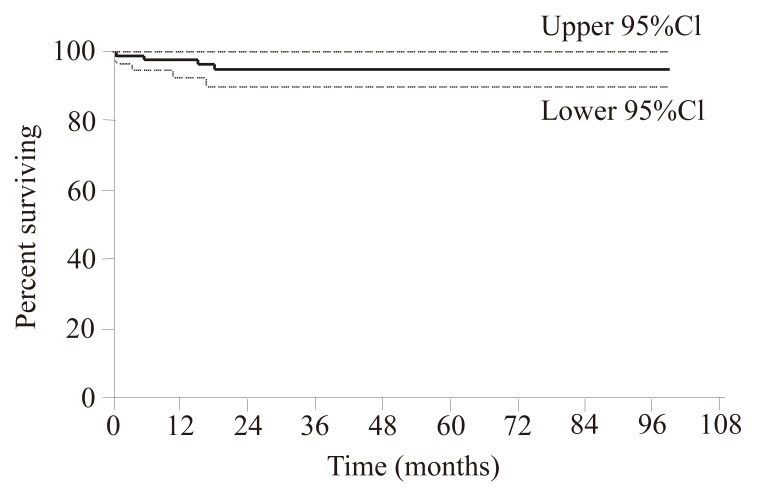
Overall survival of the 95 patients in the study group.

Progression free survival (PFS) was significantly associated with early molecular response, as was seen in the 97% of patients achieving ≥2 log reduction of their BCR-ABL transcripts after 6 months of IM therapy versus 86% in suboptimal responding patients (*p*<0.03, 95 CI, HR 7.4) (Fig. 1). Four patients (4/95, 4%) died while on treatment. The estimated OS was 98% at 1st year and 95% till 6^th^ year of FUp (Fig. 2).

Screening for ABL kinase domain mutation was performed in 15 patients (10 suboptimal responders and 5 primary resistance). All failed to achieve 2 log reductions at 6 months of IM therapy, Table 3. Mutations were detected in 11/15 (73%) of patients. Mutations in the p loop (amino acids 250–255) were found in 4/15 (27%) patients and 3 of them died. M351T was positive in 4/15 (27%) patients (one primary resistance and three suboptimal responses) and were all shifted to second generation TKI. F359V was positive in 2/15 (13%) patients with suboptimal response. Three patients (20%) were positive for Q252H, 2 primary resistant progressed to ABC and cytogenetic clonal evolution with acquisition of a new Philadelphia chromosome and the third patient acquired additional Inv 3 (q21-q26). One suboptimal responder was shifted to a second generation TKI. Y253F was found in one patient (7%) and T315I was positive in another patient (7%) and both died after progression to ABC No ABL mutation was detected among any of 30 tested IM responding patients (*p*<0.001).

**Table 3:** Correlation between mutation rate and 2 log reduction value of BCR-ABL transcripts at 6 months

2 log reduction at 6months	Mutation rate

No (15 patients)	11/15 (73%)
Yes (30 patients)	0/30 (0%)

*p*<0.001.

## DISCUSSION

Imatinib mesylate is, at present, the standard therapy for CML, and this therapy results in rapid hematological and cytogenetic responses in the majority of patients. The International Randomized Study of Interferon versus STI571 (IRIS) study demonstrated the efficacy of this drug in conferring hematologic, cytogenetic and molecular responses in CPCML patients as well as favorably impacting on survival (7). In the present study we focused on the value of early molecular response as a predictor of longer PFS and of sustained hematological and cytogenetic response. Patients who achieved ≥2 log reduction of BCR-ABL transcripts at 6 months after initiation of IM 400 mg daily showed a significantly higher rate of CCyR at 12 month, MMR at 18 months, as well as longer PFS. Patients who attained this log reduction level had a significantly shorter median time to attain CCyR. Molecular response as early as after 6 months of 400 mg IM daily could be thus considered a significant prognostic cut off value. This finding was consistent with a previous study where the dynamics of BCR-ABL transcripts - earlier at 2 months of IM therapy - were used to predict cytogenetic response at 6 months (17). However, we observed that only 26% of patients achieving >2 log reduction after 6 months of treatment did not show CCyR. Some studies related this phenomenon to a faster down regulation of the BCR-ABL fusion gene by IM (17) while other studies explained it by the higher mitotic activity of residual CML cells detected by cytogenetic analysis (18). We consider the delay in the initiation of IM therapy after diagnosis, and the likely prior exposure to other therapies, as potential additional factors behind this heterogeneity in responses in our patients. In this study we used the standard dose of 400 mg IM daily monotherapy, and 45% of the entire research cohort group attained MMR at 12 months, reaching 64% at 18 months, which was in close conformity with the IRIS study, and with other studies (5–7). We also found a strong association between cytogenetic and molecular responses. The achievement of a ≥2 log reduction value after 6 months of treatment had also a significant association with the type of response, and could discriminate between optimal, and suboptimal responders from resistant cases. Depending on this cut off value, the screening for mutations in the ABL domain could be implemented earlier in the course of treatment, permitting either IM dose escalation or treatment modification. All patients showing primary resistance, and 89% of suboptimal responders, failed to achieve 2 log reductions by 6 months versus only 3% of the optimal responder group (*p*<0.0001), indicating that this cut off molecular value carries a prognostic implication for selecting patients for mutations testing. The ABL kinase domain mutation rate among this group was significantly higher than among optimal responding patients. Point mutation within ABL kinase domain has been reported as a common mechanism of resistance (19). Progression to ABC was also regarded as a predictive finding for mutation as the 3 out of 5 tested resistant patients who died were found to harbor a mutation in the p-loop (2 patients with Q252H and one patient with Y253F). This finding agrees with the possibility that some p loop mutations increase the oncogenicity of BCR-ABL (19). Of the 10 mutations tested, M351T was the more commonly detected (27%) followed by Q252H (20%) which is in accordance with previous study (19). Progression free survival (PFS) at 18 months was 95%. The median survival after 6 years was 95%. Whether these results will be maintained will be known only with longer follow up. It has been recently demonstrated that the achievement of MMR at any time during therapy was a significant and independent predictor of PFS (6). The present study demonstrated a significant association between the molecular response after 6 months of IM monotherapy with longer PFS, which consolidates previous reports that molecular responses are predictors of prolonged PFS or predictors of acquisition of resistance (9). We also suggest that ≥2 log reduction in BCR-ABL transcripts at 6 months is an earlier prognostic marker for stratifying patients for their risk of progression, as well as for stratifying patients for consideration of IM dose escalation or other alteration in therapy such as switching to second generation TKI drugs. Additional evidence for this comes from the observation that all patients who progressed to ABC failed to achieve two log reductions at 6 months.

In conclusion, for IM–treated patients with CPCML, the routine clinical use of sequential molecular monitoring may allow prediction of the disease course and helps risk stratification. Failure to achieve the response level of 2 log reduction at 6 months could be added to the criteria of definitions of late, suboptimal response, or resistance and may point in due time to the need for ABL domain kinase mutation testing in view of therapy modification.
